# The effect of exercise on baseline SCAT5 performance in male professional Rugby players

**DOI:** 10.1186/s40798-020-00265-8

**Published:** 2020-08-15

**Authors:** Ross Tucker, James Brown, Eanna Falvey, Gordon Fuller, Martin Raftery

**Affiliations:** 1 World Rugby, World Rugby House 8-10 Lower Pembroke Street, Dublin 2, Ireland; 2 Department of Orthopaedics, Institute of Sport and Exercise Medicine, Stellenbosch University, Tygerberg, 7500 South Africa; 3 Department of Medicine, University College Cork, Cork, Ireland; 4 Centre for Urgent and Emergency Care Research, School of Health and Related Research, University of Sheffield, Sheffield, UK

**Keywords:** Concussion, SCAT, Rugby union, Neurological screening, Concussion management

## Abstract

**Background:**

Rugby Union requires annual baseline testing using the Sports Concussion Assessment Tool (SCAT5) as part of its head injury assessment protocols. Scores achieved during baseline testing are used to guide return-to-play decisions at the time of head impact events during matches, and concussion diagnosis during subsequent diagnostic screens. Baseline values must be valid, accurate representations of a player’s capability in the various SCAT5 sub-modes, including symptom report, cognitive function and balance. The extent to which prior exercise may affect performance is an important consideration, and the present cross-sectional study aimed to explore how SCAT5 performance differs when assessed at rest (RSCAT) compared to after 30 min of exercise (EXSCAT) in 698 male professional rugby players for whom paired exercise and rest SCAT5 data were available.

**Results:**

Symptom endorsement was greater when assessed after exercise than at rest. Fatigue/Low energy was 1.5 times more likely to be reported when assessed during EXSCAT. Orientation score was improved during SCAT5s performed after exercise, but only when rest and exercise SCAT5s were conducted on the same day, suggesting a learning effect. Concentration score was impaired during EXSCAT. No other cognitive sub-modes were affected by exercise. Total errors during Modified Balance Error Scoring System (MBESS) increased during EXSCAT, as a result of increased errors made during single leg balance, irrespective of testing sequence, with 42% of players making more errors in EXSCAT, compared to 28% making more errors in RSCAT.

**Conclusions:**

Symptoms, cognitive sub-modes and balance sub-modes are all affected by exercise. These may be the result of learning effects that improve cognitive performance, and the direct effects of exercise on sub-mode performance. The clinical implications of these changes may be assessed in the future through a study of diagnostic screens in players after head impact events, to confirm whether an exercise baseline screen is required annually, or whether specific sub-modes of the SCAT5 should be obtained at rest and after exercise.

## Key points

- SCAT5 assessments undertaken after exercise in elite male rugby players show significant differences in symptoms, cognitive sub-test performance and balance sub-test performance.
- Symptom endorsement is higher when assessed immediately after exercise compared to at rest, while balance errors increase after exercise.
- Cognitive function may be influenced by learning effects when exercise and resting SCAT5s are undertaken in close proximity to one another, which has implications for the order of testing and gap between tests in order to maximize validity.
- More research is required to adequately explore the clinical implications of these exercise-induced changes to baseline performance, with specific focus on diagnostic settings after head impact events.

## Introduction

The Sports Concussion Assessment Tool (SCAT) was first developed in 2004 after the 2nd international conference on concussion [1] using tests from eight existing tools, as a standardised assessment tool for acute concussion, with the most recent iteration being the SCAT5 [2].

Rugby Union implemented the SCAT in the professional game as part of a comprehensive Head Injury Assessment process for management of head impact events occurring during matches. An abridged form of the SCAT is used as an in-game off-field assessment; subsequently, the complete SCAT5 is used during diagnostic assessments performed within three hours of the head impact event (HIA2 screen) and after two nights’ rest (HIA3 screen) [3].

World Rugby requires mandatory annual completion of a baseline SCAT in professional players, usually performed in the pre-season, with subsequent screening and diagnostic results evaluated relative to these uninjured baseline results. In the absence of baseline testing, normative data, derived from a valid and large comparable dataset, may be used to identify a clinical reference limit that supports return to play decisions and concussion diagnosis [4].

The validity of baseline testing results is thus an important component of concussion management. Various factors affecting baseline performance and clinical assessments after concussion have been explored, including differences in baseline performance by sex and age [5], post-concussion results assessed against baseline in men and women [6] and the effects of a previous concussion on resting (baseline) assessments [7–10].

An important consideration is the effect of exercise on baseline performance, given that SCAT assessments will be conducted during matches as part of the concussion screening tool. Were exercise to influence baseline performance, it would have implications for what is considered abnormal at the time of screening after head impacts, so it has recently been proposed that screen validity would improve if baseline testing is conducted immediately after exercise [2, 11, 12]. Gaetz et al. found that a bout of mild exercise (15 min cycle ergometry) increased balance problems, numbness and tingling when compared to a pre-exercise baseline test [13]. Fatigue, induced by aerobic and anaerobic exercise, was found to impair postural control in healthy athletes [14], and Lee et al. found impaired balance sub-test performance, and greater symptom endorsement when assessed after exercise [15].

Therefore, given these findings, it is prudent for World Rugby to consider whether the same findings exist in elite professional male players.

### Aims

The aim of the present study was to explore the effects of exercise on subsequent SCAT5 performance. We assess whether symptoms reported, and cognitive and balance sub-mode performances are different when baseline testing is undertaken after exercise compared to at rest. We explore whether SCAT5 performance during exercise and rest is affected by testing order, namely whether a resting assessment is conducted before or after an exercise assessment, and whether any possible differences between exercise and rest assessments are affected by the days between tests. These findings have clinical implications for whether annual baseline testing should include an exercise assessment for sub-mode comparisons during off-field screening assessments, and the order and spacing between tests. We also aimed to identify whether the thresholds for abnormal sub-mode results using normative data should be adjusted for tests that are conducted immediately after exercise.

## Methods

### Study design, setting and study population

A cross-sectional study was performed using data from the World Rugby Head Injury Assessment (HIA) database, which contains baseline, match-day off-field concussion screening results and diagnostic assessments from the professional game. In order to use the HIA process, a competition must adhere to mandatory competition player welfare standards [World Rugby Player Welfare Site] that ensures a standardised approach to concussion detection and management as well as data collection. The source population thus comprises the majority of eligible professional male players in domestic and international competitions who underwent mandatory baseline SCAT assessments between 2015 and 2019. All data are de-identified prior to exporting, with anonymized unique IDs to match cases for subsequent analysis.

### Baseline screening

The SCAT assessments were administered prior to commencement of the relevant competition season or tournament, according to methods described previously [16]. Within this cohort, a group of men’s professional players with paired SCAT5 at rest (RSCAT) and SCAT5 immediately after exercise (EXSCAT) was identified for analysis of the effect of prior exercise on baseline SCAT performance. Paired tests were conducted within 2 weeks of one another.

The cohort of paired exercise and resting tests was divided into two groups who performed their EXSCAT or RSCAT in different orders. These groups are considered separately in subsequent analyses. For some sub-modes, changes to the SCAT5 were made during the sampling period, including the adoption of a 10-Word rather than 5-Word list for Immediate Memory and Delayed Recall. Available case analyses were subsequently performed including only paired cases with identical test versions. Where sample numbers for paired data were affected by this change, the sample size is indicated for each analysis.

EXSCAT was performed immediately after a bout of high intensity exercise bike protocol. The protocol comprised 30 min of cycling above 80% of the age-predicted maximum heart rate, and reach a target of 7 or greater on the 10-point Rating of Perceived Exertion scale prior to undertaking the EXSCAT.

For all analyses, differences between EXSCAT and RSCAT were calculated as the sub-mode score during exercise minus the sub-mode score at rest, and analysed by comparing the 95% CI, and determining the proportion of players who improved, worsened and remained the same for each sub-mode. A positive sign for sub-mode change means that the score during EXSCAT was greater than during RSCAT. For symptoms, balance tests and tandem gait, this indicates a relative worsening of sub-mode performance during EXSCAT, since more symptoms are endorsed, more errors made or greater tandem gait time during exercise compared to rest. For cognitive sub-modes, a positive sign indicates an improved score for that sub-mode during EXSCAT.

The Shapiro-Wilk test was conducted to determine whether the changes in sub-mode score was normally distributed, with a *p* value of < 0.05 indicating a rejection of the null hypothesis: normal distribution.

The potential for learning effects when two baselines are conducted in very close proximity was explored through two methods. First, the effect of testing order on the change in sub-mode scores was investigated using an ordinal regression model, with the change in sub-mode score (EXSCAT minus RSCAT) categorised as “worsened” (− 1), “no change” (0) or “improved” (+ 1) as the dependent variable; and testing order (REST1 or EX1) and time between tests (binary: 0 = same day; 1 = different day) as the independent variable. An odds ratio was calculated for each sub-mode, reporting the adjusted odds of sub-mode performance improving during EXSCAT when RSCAT was performed first. An odds ratio greater than 1 and a *p* value less than 0.0042 thus means that an improvement in sub-mode performance during EXSCAT is more likely in the REST1-EX2 testing order.

Second, the two test orders were divided into those conducted on the same day and those performed more than one day apart. Changes in sub-mode performance were then assessed as described previously.

Outlier changes in sub-mode score were investigated using Bland-Altman’s limits of agreement approach [17]. Outliers were identified as scores that changed by more than the sub-mode mean ± limits of agreement (LOA), where LOA was calculated as 1.96 × SD. A Wilcoxon rank sum test was performed to determine whether the distribution of sub-mode scores was significantly different between the paired EXERCISE and REST measurements, with the null hypothesis (EX = REST) rejected when *p* < 0.004, based on a Bonferroni correction of the original alpha of 0.05, divided by the 12 sub-domains (0.05/12 = 0.0042).

To compare the frequency of reporting of specific symptoms (e.g. headache) under the two conditions (exercise and rest), McNemar’s chi-squared analysis—for paired data—was performed. The Fisher’s exact *p* value of this test was presented if any specific comparisons had had fewer than 10 cases. As there were 22 symptoms, the Bonferroni adjusted *p* value was 0.0022 (0.05/22) for these analyses.

## Results

Six hundred and ninety-eight paired RSCAT and EXSCAT assessments made up the cohort. Within this cohort, 542 players performed RSCAT before EXSCAT (REST1-EX2 order), while 156 players performed EXSCAT first (EX1-REST2 order).

The median days between paired SCATs differed significantly between the two testing orders (Kruskal-Wallis *p* < 0.0001). For the REST1-EX2 testing order, the median days between SCATs was 0.03 days (interquartile range 0.02–1.09), with 375 (67%) of the EXSCAT and RSCAT assessments conducted on the same day. In contrast, when EXSCAT was performed first (EX1-REST2), median days between testing was 3.03 (IQR 0.96–8.87), with only 18% conducted on the same day, and 33% separated by more than 1 week (Table 1).

**Table 1:** Summary of sub-mode performances during SCAT5s at rest (RSCAT) and immediately after exercise (EXSCAT)

		Resting SCAT performed first, REST1-EX2 (*n* = 542)				Exercise SCAT performed first, EX1-REST2 (*n* = 156)			
Median days between tests		0.03 days (IQR 0.02–1.09)				3.03 days (IQR 0.96–8.87)			
		RSCAT		ExSCAT		ExSCAT		RSCAT	
	Scale	Mean (SD)	Median	Mean (SD)	Median	Mean (SD)	Median	Mean (SD)	Median
Symptom number	0–22 points	1.11 (2.42)	0	1.21 (2.59)	0	1.23 (2.56)	0	0.53 (1.5)	0
Symptom severity	0–132 points	1.64 (4.26)	0	1.78 (4.2)	0	1.94 (4.69)	0	0.71 (2.06)	0
Orientation	0–5 points	4.83 (0.38)	5	4.88 (0.33)	5	4.82 (0.41)	5	4.79 (0.42)	5
Immediate memory (*n* = 424 Rest1; 63 Ex1)	0–30 points	21.63 (3.79)	22	21.65 (3.99)	21	21.34 (3.91)	21	20.96 (3.74)	21
Concentration	0–5 points	4.17 (0.98)	5	4.22 (0.94)	5	4.21 (1.04)	5	4.42 (0.85)	5
Delayed recall (*n* = 424 Rest1; 63 Ex1)	0–10 points	6.93 (1.9)	1	6.95 (2.09)	1	7.22 (1.8)	3	6.82 (2)	1
Tandem gait assessment	seconds	10.58 (1.62)	11	10.06 (1.56)	10	10.27 (1.74)	10.15	10.19 (1.71)	10
M-BESS									
Double leg	Errors made	0.02 (0.25)	0	0 (0.11)	0	0.01 (0.16)	0	0.01 (0.11)	0
Single leg	Errors made	2.07 (1.96)	2	2.42 (2.01)	2	2.4 (2.07)	2	1.91 (1.62)	2
Tandem stance	Errors made	0.79 (1.2)	0	0.95 (1.29)	0	0.83 (1.27)	0	0.71 (1.17)	0
Total errors	Errors made	2.89 (2.55)	2	3.38 (2.75)	3	3.25 (2.8)	3	2.64 (2.24)	2

**Fig. 1:**
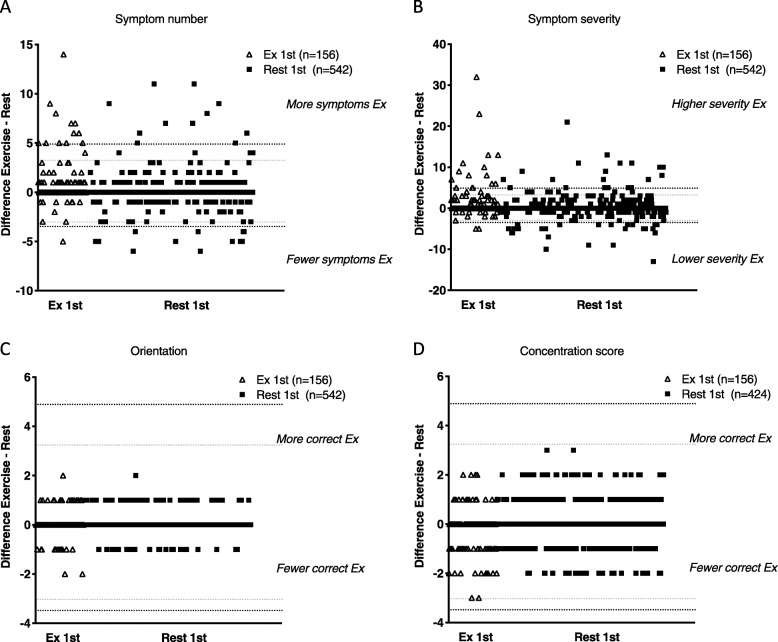
Differences in symptom and cognitive sub-domain scores between EXSCAT and RSCAT **a**-**d**. Open triangles indicate players who performed EXSCAT first, and solid squares represent players who followed the REST1-EX2 testing sequence. The horizontal dashed lines indicate the Mean change ± LOA for each sub-mode and testing sequence, with EXSCAT first indicated by the black lines and RSCAT first by the grey lines

**Fig. 2:**
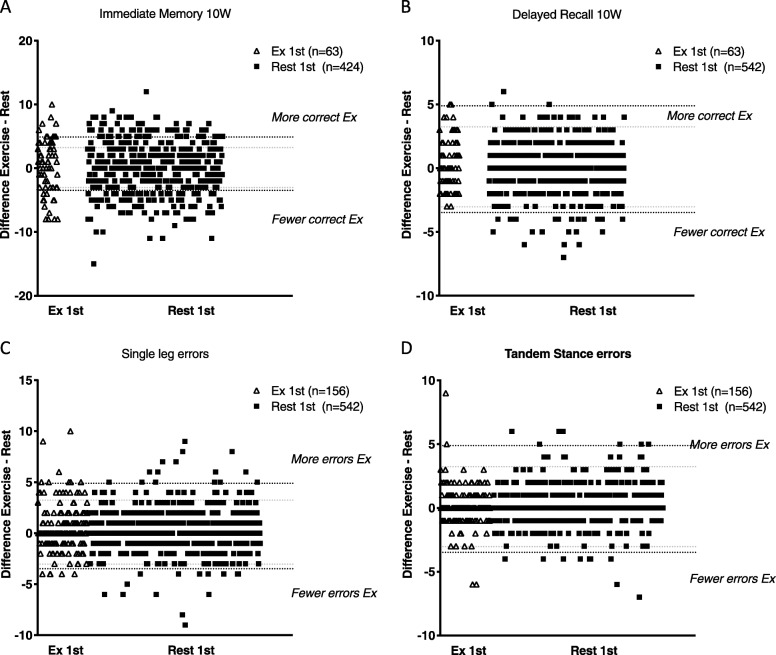
**a**-**d** Differences in memeory and balance sub-domain scores between EXSCAT and RSCAT. Open triangles indicate players who performed EXSCAT first, and solid squares represent players who followed the REST1-EX2 testing sequence. The horizontal dashed lines indicate the Mean change ± LOA for each sub-mode and testing sequence, with EXSCAT first indicated by the black lines and RSCAT first by the grey lines

Differences in selected sub-domain scores between EXSCAT and RSCAT are plotted in Figs. 1 and 2.

Table 2 shows the difference in sub-mode scores, calculated as score during EXSCAT minus score during RSCAT

**Table 2:** Changes in sub-mode score and evaluation of sub-mode performance

Submode	Test condition	*n*	Change in sub-mode score (Ex–rest, mean (95% CI)	Exercise vs. rest change	LOA	Improved (%)	Worse (%)	No change (%)	Improvement above LOA (%)	Worsened below LOA (%)	Wilcoxon rank sum test *p* value	Shapiro-Wilk test of normality of change score	Odds ratio (95% CI) for EX changing compared to REST when REST is assessed first
Symptom number	*Rest first (REST1-EX2)*	542	0.1 (− 0.03–0.23)	No change	3.13	14%	16%	70%	2%	3%	0.1368	**<0.001**	**2.29 (1.50**–**3.48),** ***p*** **< 0.001**
	*Exercise first (EX1-REST2)*	156	0.7 (0.37–1.04)^a^	**Worse**	4.18	7%	28%	65%	1%	8%	< **0.001**		
Symptom severity	*Rest first*	542	0.13 (− 0.07–0.34)	No change	4.80	17%	17%	65%	3%	4%	0.5933	< **0.001**	**2.21 (1.47**–**3.33),** ***p*** < **0.001**
	*Exercise first*	156	1.23 (0.6–1.86)^a^	**Worse**	7.88	8%	28%	63%	0%	4%	< **0.001**		
Orientation	*Rest first*	542	0.04 (0.01–0.07)^a^	**Improve**	0.71	9%	4%	87%	9%	4%	0.0026	0.099	0.94 (0.54–1.66), *p* = 0.842
	*Exercise first*	156	0.02 (− 0.05–0.1)	No change	1.04	13%	10%	78%	1%	1%	0.641		
Immediate memory	*Rest first*	424	0.01 (− 0.37–0.4)	No change	8.09	45%	42%	13%	0%	2%	0.7589	0.258	0.76 (0.43–1.31), *p* = 0.319
	*Exercise first*	63	0.38 (− 0.67–1.44)	No change	8.41	54%	38%	8%	2%	0%	0.4024		
Delayed recall	*Rest first*	424	0.01 (− 0.18–0.21)	No change	4.04	39%	37%	24%	1%	2%	0.5453	0.471	0.95 (0.56–1.63), *p* = 0.864
	*Exercise first*	63	0.39 (− 0.1–0.89)	No change	3.98	41%	37%	22%	3%	0%	0.2633		
Digits backwards	*Rest first*	542	0.03 (− 0.03–0.1)	No change	1.63	22%	19%	59%	5%	4%	0.2848	< **0.001**	1.55 (1.05–2.29), *p* = 0..026
	*Exercise first*	156	− 0.17 (− 0.31–-0.04)	No change	1.67	13%	25%	62%	3%	8%	0.013		
Concentration	*Rest first*	542	0.04 (− 0.02–0.12)	No change	1.70	23%	20%	57%	6%	4%	0.2827	**0.011**	**1.77 (1.20**–**2.60),** ***p*** **= 0.004**
	*Exercise first*	156	− 0.21 (− 0.34–-0.07)^a^	**Worse**	1.71	13%	28%	59%	3%	8%	0.0043		
Tandem gait	*Rest first*	541	− 0.51 (− 0.62–-0.39)^a^	**Improve**	2.72	50%	22%	28%	1%	1%	< **0.0001**	**0.005**	1.69 (1.16–2.44), *p* = 0.006
	*Exercise first*	156	0.08 (− 0.16–0.33)	No change	3.11	35%	33%	33%	1%	3%	0.7602		
Double leg balance	*Rest first*	542	− 0.01 (− 0.03–0.01)	No change	0.55	1%	1%	98%	1%	1%	0.5245	< **0.001**	1.18 (0.28–4.96), *p* = 0.824
	*Exercise first*	156	0 (− 0.03–0.03)	No change	0.39	1%	1%	98%	1%	1%	0.5686		
Single leg balance	*Rest first*	542	0.34 (0.17–0.51)^a^	**Worse**	4.07	28%	43%	29%	3%	3%	< **0.0001**	< **0.001**	0.80 (0.56–1.15), *p* = 0.233
	*Exercise first*	156	0.48 (0.12–0.84)^a^	**Worse**	4.47	29%	38%	33%	3%	5%	**0.0268**		
Tandem stance balance	*Rest first*	542	0.15 (0.02–0.27)	No change	2.91	22%	28%	51%	3%	3%	**0.0086**	< **0.001**	0.80 (0.55–1.17), *p* = 0.255
	*Exercise first*	156	0.12 (− 0.11–0.37)	No change	3.08	25%	29%	46%	4%	1%	0.1519		
Total balance score	*Rest first*	542	0.48 (0.26–0.7)^a^	**Worse**	5.09	29%	44%	27%	2%	3%	< **0.0001**	< **0.001**	0.80 (0.55–1.16), *p* = 0.229
	*Exercise first*	156	0.61 (0.13–1.09)^a^	**Worse**	6.04	32%	44%	24%	2%	3%	**0.0115**		

The relative performance change (EXSCAT – RSCAT) is indicated for each sub-mode and testing condition (EXSCAT or RSCAT performed first). *denotes significant difference EXSCAT vs RSCAT. The LOA is indicated for sub-mode and testing order. The Wilcoxon rank sum test *p*-value signifies differences between RSCAT and EXSCAT for that testing order. The odds ratio shows the odds (95% CI) of exercise performance improving relative to rest when tested in the REST1-EX2 order

^a^Adjusted for gap between days in subsequent SCAT, categorised as within the same day (0), and on a different day (1)

Symptom number and severity were greater during EXSCAT when performed first (increase of 0.7 (0.37–1.04) symptoms for EX1-REST2). Twenty-eight percent of players reported more symptoms after EXSCAT, compared to 7% who reported fewer symptoms. No differences in symptom number or severity were found for REST1-EX2. The odds of reporting fewer symptoms during EXSCAT when RSCAT was assessed first were 1.92 (1.26–2.93), indicating greater symptom endorsement during EXSCAT when conducted first.

Orientation score improved during EXSCAT, but only when RSCAT was performed first. Digits backwards and concentration score (comprised of Digits backwards and months in reverse) tended to be worse during EXSCAT when performed before RSCAT, but this did not reach significance (decrease in concentration score of − 0.21 (− 0.07 to − 0.34 for EX1-REST2). There was no testing order effect on the change in cognitive sub-modes, as indicated by the odds ratios in Table 2.

Single leg balance errors increased irrespective of testing order, with increases of 0.34 for EX1-REST2 and 0.48 for REST1-EX2 (Table 2). Tandem stance errors were greater during EXSCAT for the REST1-EX2 testing order. As a result, total errors made was greater for EXSCAT in both testing orders. Tandem gait time was faster during EXSCAT for the REST1-EX2 first testing order, with a tendency for a significant order effect for Tandem Gait time to improve more during EXSCAT for REST1-EX2 (odds ratio 1.69 (1.16–2.44), *p* = 0.006).

To explore whether the period between paired tests may influence these findings, we compared tests completed on the same day (SAME, *n* = 375 for REST1-EX2 and *n* = 28 for EX1-REST2) to those completed more than 1 day apart (DIFF, *n* = 167 for REST1-EX2 and *n* = 128 for EX1-REST2). Table 3 presents a summary of the statistical comparisons between all EXSCAT vs. RSCAT for each testing order (as per Table 2), and compares SAME and DIFF day SCATs to determine whether the difference in sub-mode scores persists when the period between tests is either shorter or longer.

**Table 3:** EXSCAT vs RSCAT sub-mode performance comparisons for test order and period between tests

	Rest first, REST1-EX2 order			Exercise first, EX1-REST2 order		
	Overall EX vs. REST (*n* = 542)	Same day (*n* = 375)	Different day (*n* = 167)	Overall EX vs. REST (*n* = 156)	Same day (*n* = 28)	Different day (*n* = 128)
Symptom number	No difference	No difference 0.025	No difference	**More symptoms** < **0.001**	No difference	**More symptoms** < **0.001**
Symptom severity	No difference	No difference	No difference	**Increased severity** < **0.001**	No difference	**Increased severity** < **0.001**
Orientation	**Improved score 0.0026**	**Improved score** < **0.001**	No difference	No difference	No difference	No difference
Immediate memory	No difference	No difference	No difference	No difference	No difference	No difference
Delayed recall	No difference	No difference	No difference	No difference	No difference	No difference
Digits backward	No difference	No difference	No difference	No difference 0.016	No difference	No difference 0.009
Final concentration	No difference	No difference	No difference	No difference 0.005	No difference	No difference 0.005
Tandem gait	**Improved time** < **0.001**	**Improved time** < **0.001**	**Improved time** < **0.001**	No difference	No difference	No difference
Double leg balance	No difference	No difference	No difference	No difference	No difference	No difference
Single leg balance	**More errors** < **0.001**	**More errors 0.001**	No difference 0.008	No difference	No difference	No difference
Tandem stance balance	No difference 0.029	No difference	**More errors 0.003**	No difference	No difference	No difference
Total balance errors	**More errors** < **0.001**	No difference 0.008	**More errors 0.002**	No difference 0.033	No difference	No difference 0.018

Calculated *p* values indicate whether sub-mode performance differed between EXSCAT and RSCAT when conducted within one day (same day) or on a different day

Symptom number and severity were elevated during EXSCAT compared to RSCAT when performed first and on different days. No differences were found when RSCAT was performed first, regardless of whether EXSCAT was conducted on the same or a different day (Table 3). Orientation score was higher during EXSCAT for the REST1-EX2 testing order, but only when tests were done on the same day. No differences were found for other cognitive sub-modes, though Digits Backward and Final Concentration tended to be impaired during EXSCAT when assessments were done on different days (*n* = 128) and overall (*n* = 156).

For the REST1-EX2 testing order, single leg balance errors were greater during EXERCISE SCAT only when tests were on the same day. Total balance errors were not different between EXSCAT and RSCAT with the SAME and DIFF when tests were on different days.

Table 4 presents the chi-squared analysis of symptoms reported during EXSCAT and RSCAT (*p* < 0.0022: 0.05/22 symptoms).

**Table 4:** Proportion of EXSCAT and RSCAT baseline assessments with symptoms endorsed

	Rest performed first (*n* = 542)			Exercise performed first (*N* = 156)		
	% of cases reported REST	% of cases reported EX	McNemar’s chi square (*exact *p* value)	% of cases reported REST	% of cases reported EX	McNemar’s chi square (*exact *p* value)
Physical	17	17	0.7681	13	19	0.0525
Neck Pain	13	10	0.0026	10	13	0.2266
Headache	6	5	0.4795	6	5	1.0000
Pressure in head	4	7	0.0046	3	7	0.0923
Nausea or vomiting	1	2	0.3018	0	4	0.0312
Fatigue or low energy	17	24	0.0001*	11	25	0.0001*
Cognitive	17	15	0.2382	6	13	0.0018*
Don’t feel right	3	3	1.000	3	6	0.0625
Difficulty concentrating	7	10	0.0326	3	9	0.0039
Difficulty remembering	10	8	0.223	3	5	0.1250
Confusion	1	2	0.0703	0	2	0.2500
Drowsiness	5	4	0.5413	1	2	0.5000
Feeling slowed down	4	6	0.0396	2	8	0.0117
Feeling like in a fog	1	2	0.2668	0	2	0.2500
Vestibulo-ocular	8	11	0.0641	4	9	0.0215
Dizziness	2	5	0.0125	1	6	0.0078
Blurred Vision	3	3	1.0000	2	3	1.0000
Balance problems	3	5	0.0081	1	4	0.1250
Sensitivity to light	4	3	0.2668	2	3	1.0000
Sensitivity to Noise	1	2	0.4531	0	1	0.5000
Psychological	15	13	0.1282	8	17	0.0026
Trouble sleeping	10	8	0.0525	6	8	0.5078
Nervousness or anxiousness	6	6	0.6291	4	6	0.4531
More emotional than normal	3	4	0.4545	1	5	0.0312
Irritability	6	5	1.0000	3	7	0.0654
Sadness	3	2	1.0000	3	4	1.0000

Calculated p-values indicate whether sub-mode performance differed between EXSCAT and RSCAT. *symptom endorsed significantly more frequently during EXSCAT within the identified test order

Cognitive symptoms were more likely to be reported when EXSCAT was performed first (*p* = 0.0018). ‘Fatigue or low energy’ was reported 1.5 times more frequently during EXSCAT than RSCAT, and was reported more frequently during EXSCAT in both orders. ‘Feeling slowed down’ (1.8 times more frequent during EXSCAT), ‘Dizziness’ (2.6 times more frequent during EXSCAT) and ‘Difficulty concentrating’ tended to be more likely to be reported after exercise, irrespective of test order.

## Discussion

This study compared baseline SCAT performance in professional rugby players under conditions of rest and immediately after exercise. We found that a number of sub-mode performances are affected by exercise, with some worsening and others improving during a SCAT conducted immediately after exercise compared to after rest.

A secondary aim of this study was to explore potential direct exercise and learning explanations for any observed changes, though we acknowledge that we cannot comprehensively evaluate the possibilities within the current study design. We looked at test order, and the duration between tests (Table 3). Our analysis raises insights regarding possible learning and exercise effects that may account for some findings, and which may be explored in specific studies in the future.

### Symptom sub-modes

The higher endorsement of symptoms during EXSCAT compared to RSCAT was only observed for the EX1-REST2 testing order (Fig. 1, Table 2) and may be the result of players reporting more symptoms during exercise as a result of acute sensations of fatigue and physical discomfort. This has previously been observed during exercise [18], during a SCAT3 conducted after a 5-min exercise bout in Rugby League players [15] and in college athletes assessed within 10 min of completing an exercise protocol [19].

Of interest is that the instruction provided to players during SCAT5s, both at rest and exercise, is to report ‘trait symptoms’, or how they typically feel. In principle, this should not be affected by exercise, since exercise would influence their ‘state’ (how they currently feel), rather than trait (which is by nature historical or recalled retrospectively). The study by Lee et al., using SCAT3, did find an increase in symptoms after exercise, but this is perhaps unsurprising given the SCAT3 requirement to report ‘state’ symptoms, which the authors proposed are induced by exercise [15]. Our finding suggests that players and potentially clinicians interpret the instruction differently, allowing exercise to increase symptoms endorsement in what appears to be a form of ‘state’ reporting. Current state, particularly fatigue, thus impacts on reported trait symptoms during EXSCAT, but not when the player is consciously aware of their very recently (SAME day) previous symptom report conducted during RSCAT. This reinforces that the symptom list used during SCAT5 is not specific to concussion [12], and thus symptoms endorsed during off-field screens during matches must be interpreted with caution.

Players were more likely to endorse symptoms during EXSCAT when this test was performed second. This test order most often occurred with same-day test completion. This may condition the player to report their trait symptoms, or how they typically feel, as per the SCAT5 instructions [2], in a manner consistent with their first test (REST1). However, when performing EXSCAT first, no such anchoring effect exists, potentially resulting in an increase in both symptom number and severity for EXSCAT (Table 3).

### Cognitive sub-modes

For cognitive tests, learning or memory effects may explain some of the differences between EXSCAT and RSCAT. We found that Orientation score was better in EXSCAT, but only in the REST1-EX2 order (Table 2, Fig. 1), and only when the tests were done on the same day (Table 3). This may suggest a learning or memory effect. During REST1, the Orientation question most frequently answered incorrectly is ‘Date’ (83 cases, 92% of the incorrect Orientation answers). During EX2, of these 83 players who incorrectly answered Date during REST1, 43 corrected their error during EX2. This accounts for 90% of the players who increased orientation score from REST1 to EX2 (Table 2), suggesting an immediate learning effect.

More complex interactions between exercise and learning may affect other cognitive sub-modes. We found that Digits Backward and Concentration performance were impaired in EXSCAT, but only for EX1-REST2. No differences between EXSCAT and RSCAT were found for Immediate Memory or Delayed Recall sub-modes (Fig. 2). It is challenging, however, to discern between the potentially conflicting effects of exercise and learning on these cognitive modes. That is, it is possible that exercise impairs cognitive sub-mode performance, which would result in lower scores in these sub-modes, as we found for Digits Backwards during EX1 compared to REST2. This may be related to the documented increase in ‘Difficult Concentrating’ during EXSCAT.

A learning effect may also exist however, which would potentially improve performance during the second assessment when compared to the first, particularly when done on the same day, as happened more often for REST1-EX2 than EX1-REST2. These two mechanisms may thus cancel one another out, and while we attempted to explore this through the division of tests into cohorts by time period (Table 3), we acknowledge that our sample size is reduced significantly, and the study design is unable to answer these questions, undermining the conclusions that may be drawn from cognitive sub-mode analyses.

### Balance sub-modes

We found that single leg balance errors increased irrespective of test order, and tandem leg stance errors were higher for REST1-EX2. The result was an overall increase in total errors during exercise in both testing orders, confirming what has been shown previously in various populations with various study designs in which balance is assessed shortly after exercise [15, 19–22].

The overall impairment of balance sub-modes during EXSCAT for both REST1-EX2 and EX1-REST2 orders cannot be attributed to a learning or practice effect, because this would predict an improvement in the second test, irrespective of test type. We therefore conclude that balance is impaired directly by exercise, perhaps as a result of fatigue, with SAME day pairings showing greater effects, potentially implicating fatigue from testing. Schneiders et al. previously found that decrements in balance performance disappeared when balance was assessed more than 20 min after exercise, supporting that the direct effects of fatigue impair balance performance.

In contrast with previous research [15], we find that the Tandem Gait assessment was completed faster in EXSCAT, but only when RSCAT preceded the EXSCAT trial (Table 2).

### Clinical implications

We document a number of sub-modes where there were statistically significant differences in performance, though these changes were often small and may have questionable clinical significance. This may be particularly the case for balance sub-modes, which have been documented to have poor repeatability and inter-rater reliability [23], and for cognitive sub-modes, given that we cannot quantify or precisely determine whether learning effects in REST1-EX2 may cancel out any possible detrimental effects on sub-mode performance during an EXSCAT. To perform the necessary sub-mode analysis, while identifying a 5% clinical reference limit for abnormal scores requires a larger sample size, with greater control over potential confounding variables such as testing order, days between tests, footwear and exercise protocol than could be provided by the present study.

Because of these limitations, we do not recommend changes to previously described clinical guidelines based on the present results. We have previously proposed clinical reference limits for each sub-mode based on 13,479 resting baseline SCATs in professional rugby players, and these should remain in place pending further research that specifically overcomes these limitations.

Such an approach may also be clinically conservative for concussion management during matches. Because false negative screening results present a clear player-safety concern, current data does not support raising number of permissible errors. These scenarios can be examined only when clinical data and off-field screens are modelled in the future.

Further research may also examine the learning effects we discuss here, by performing two resting SCAT5 assessments within a week of one another, to discern whether cognitive sub-modes in particular may be affected by learning, and to control for any possible effects of test proximity on the results.

### Limitations

The present study is unable to differentiate between mechanism of learning and possible direct effects of exercise on sub-mode performance, as described. As a result of testing order differences, and the difference in days between tests, where many tests are done on the same day, we cannot account for how immediate learning effects affect performance in the second test. While we have attempted to explore this (Table 3), this is a recognized limitation. The exercise protocol used, consisting of cycling exercise, may not accurately reflect the exercise type that would be experienced during a rugby match, which may have implications for intensity, local muscle fatigue, proprioception and thus sub-mode performance changes observed here.

## Conclusion

In conclusion, we find that symptom endorsement is greater after EXERCISE, but only when exercise baselines are performed before resting baseline assessments. Cognitive sub-modes may be affected by learning, particularly when two baseline assessments are performed in close proximity to one another, but the possible effects of exercise on cognitive function may cancel out this learning benefit, resulting in an interaction of mechanisms that may be explored during future studies. Balance testing, particularly during single leg stance, is compromised when assessed after exercise compared to rest. The clinical implications of these changes may be assessed in the future through a study of diagnostic screens in players after head impact events, to confirm whether an exercise baseline screen is required annually, or whether specific sub-modes of the SCAT5 should be obtained at rest and after exercise. Until such research can be conducted in a valid clinical setting, we recommend that the normative reference limits for all sub-modes remain as we have previously proposed, and that sub-mode performance during return-to-play and diagnostic screens be assessed relative to a resting baseline screen, as is currently done.

## Abbreviations

SCAT: Sports Concussion Assessment Tool
HIA: Head injury assessment
